# Genomic complexity and dynamics of clonal evolution in childhood acute myeloid leukemia studied with whole-exome sequencing

**DOI:** 10.18632/oncotarget.10778

**Published:** 2016-07-22

**Authors:** Riccardo Masetti, Ilaria Castelli, Annalisa Astolfi, Salvatore Nicola Bertuccio, Valentina Indio, Marco Togni, Tamara Belotti, Salvatore Serravalle, Giuseppe Tarantino, Marco Zecca, Martina Pigazzi, Giuseppe Basso, Andrea Pession, Franco Locatelli

**Affiliations:** ^1^ Department of Pediatrics “Lalla Seràgnoli”, Hematology-Oncology Unit, University of Bologna, Bologna, Italy; ^2^ Interdepartmental Centre of Cancer Research “G. Prodi”, University of Bologna, Bologna, Italy; ^3^ Current address: Stem Cell Group, University College London Cancer Institute, University College London, London, United Kingdom; ^4^ Department of Pediatric Hematology Oncology, Fondazione IRCCS Policlinico San Matteo, Pavia, Italy; ^5^ Department of Woman and Child Health, Laboratory of Hematology-Oncology, University of Padova, Padova, Italy; ^6^ Department of Pediatric Hematology-Oncology, IRCCS Ospedale Bambino Gesù, Rome, Italy; ^7^ University of Pavia, Pavia, Italy

**Keywords:** pediatric acute myeloid leukemia, acute myeloid leukemia relapse, whole-exome massively parallel sequencing, SETD2 mutation, FLT3-TKD mutation

## Abstract

Despite significant improvement in treatment of childhood acute myeloid leukemia (AML), 30% of patients experience disease recurrence, which is still the major cause of treatment failure and death in these patients. To investigate molecular mechanisms underlying relapse, we performed whole-exome sequencing of diagnosis-relapse pairs and matched remission samples from 4 pediatric AML patients without recurrent cytogenetic alterations. Candidate driver mutations were selected for targeted deep sequencing at high coverage, suitable to detect small subclones (0.12%). BiCEBPα mutation was found to be stable and highly penetrant, representing a separate biological and clinical entity, unlike WT1 mutations, which were extremely unstable. Among the mutational patterns underlying relapse, we detected the acquisition of proliferative advantage by signaling activation (PTPN11 and FLT3-TKD mutations) and the increased resistance to apoptosis (hyperactivation of TYK2). We also found a previously undescribed feature of AML, consisting of a hypermutator phenotype caused by SETD2 inactivation. The consequent accumulation of new mutations promotes the adaptability of the leukemia, contributing to clonal selection. We report a novel ASXL3 mutation characterizing a very small subclone (<1%) present at diagnosis and undergoing expansion (60%) at relapse. Taken together, these findings provide molecular clues for designing optimal therapeutic strategies, in terms of target selection, adequate schedule design and reliable response-monitoring techniques.

## INTRODUCTION

Outcome of children with acute myeloid leukemia (AML) has improved significantly over the past 30 years [1]. Although the majority of patients reach complete remission with intensive chemotherapy [2] [3] [4], about 30% of children with AML experience disease recurrence [5] [6]. The outcome of these patients is poor, with a probability of overall survival (pOS) ranging between 29% and 38% [5] [6] [7], making relapsed AML a striking challenge and the leading cause of death in these children. Given the high frequency of treatment-related deaths (5%–10%), both with first-line treatment and with protocols for relapsed disease, further intensification of standard chemotherapy does not seem to be an option for further improvement of patients' outcome [8]. This said, better knowledge of the molecular lesions underlying AML and especially of those involved in the development of relapse is mandatory in order to devise novel patient-specific treatment strategies. Based on recent studies, disease recurrence seems to be associated with clonal evolution from early stage to relapse, promoted at least in part by chemotherapy itself. This model is supported by the seminal work of Ding et al [9] in adult AML, and has only recently been confirmed by Farrar et al [10] in childhood AML. These authors detected different subclones within the whole tumor population which were characterized by the acquisition of additional mutations. The unequal fitness for survival of various subclones provides them with different capability of escaping chemotherapy, this leading to relapse. To gain deeper knowledge on the molecular mechanisms underlying relapse, we performed whole-exome sequencing (WES) of primary tumor-relapse pairs and matched remission samples from 4 pediatric AML patients. Candidate driver somatic events were identified and selected for targeted deep sequencing, a sensitive assay capable to detect their presence throughout the various phases of the disease and to track the dynamics of evolution of the various subclones. We focused on cases without recurrent cytogenetic alterations, which represent about 20% of childhood AML. In this group of patients, adequate molecular characterization, risk stratification and disease monitoring remain difficult tasks, this leading to a great variability in terms of response to therapy and final prognosis.

## RESULTS

### Polyclonal structure and clonal evolution in childhood AML revealed by whole-exome sequencing

**Figure 1:**
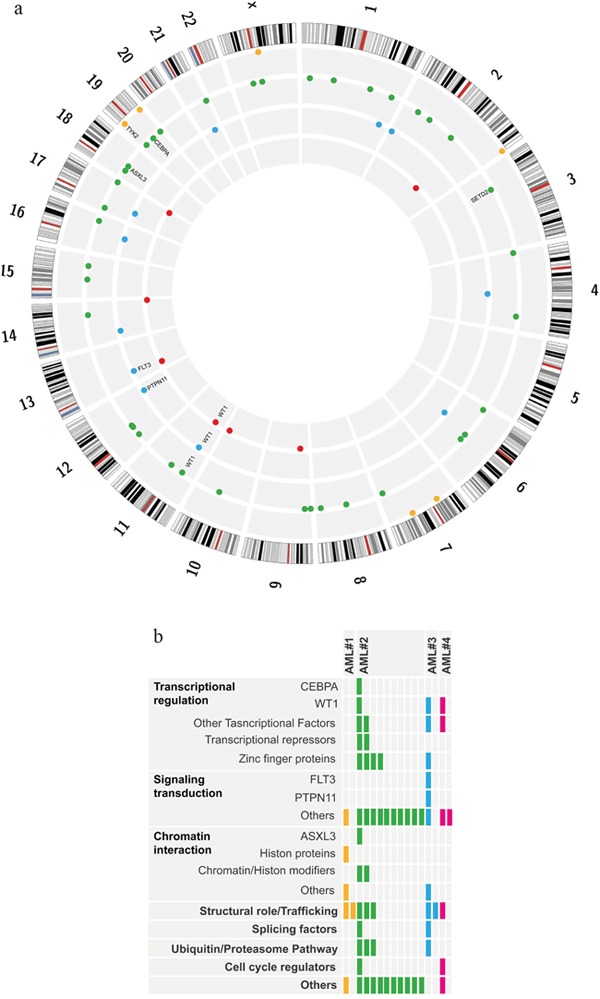
Somatic non synonymous mutations detected by WES in 4 pediatric AML **Panel 1a:** The image is a plot created with the use of Circos software (*http://circos.ca/*), showing all somatic mutations detected in each patient. Chromosomes are arranged clockwise from chromosome 1 to X, each grey circle represents a single patient, proceeding from AML#1 to AML#4 from the outer to the inner circle, each dot represents one mutation. **Panel 1b:** Mutations are grouped into functional categories of genes involved according to Pubmed annotation. Each box represents a single mutation, each color represents a distinct patient.

WES of primary tumor-relapse pairs and matched remission samples from 4 childhood AML patients (Table 1) resulted in 9.34×10^8^ reads, yielding >95% diploid exome coverage. Average haploid coverage of targeted (10X) regions for each sample was between 51X and 75X. A total amount of 65 single nucleotide variants (SNVs) and 17 insertion/deletion mutations (*Ins/Dels*) were considered somatic mutations (for detailed criteria refer to Supplementary Information), resulting in: 6 mutations in AML#1, 41 in AML#2, 11 in AML#3 and 7 in AML#4. These results agree with the evidence that the AML genome usually has a low number of somatic mutations [11], with the exception of AML#2 samples, which were found to carry a higher burden of mutations, despite comparable sequencing performance. Exome-wide single-nucleotide polymorphism analysis revealed very few copy-number events, concordantly with literature reports [11] [12], except in AML#1 sample, which carried a large region of gain (17:18965000-81188000) and a region of loss (17:0-18965000) of copy number at diagnosis. This alteration was lost at relapse, when it was no longer detectable within the sensitivity of the assay, likely due to its subclonal nature. The list of all novel somatic SNVs and *Ins/Dels* for each patient is provided in Supplementary Table S1.1-S1.4 and is summarized in Figure 1. WES allowed us to detect diagnosis-specific mutations, relapse-specific mutations and mutations shared between the primary and the relapse samples of each patient. These results confirmed that childhood AML is a polyclonal disease, with subclonal architecture of the whole leukemic population changing from diagnosis to relapse. With the attempt to backtrack the origin of such clones, we performed targeted deep sequencing of candidate driver mutations based on a comprehensive analysis of their recurrence in AML or on a possible pathogenetic role, according to literature. We obtained an average coverage of ~7000X, with an estimated sensitivity of 0.12% in detecting minor subclones. This allowed us to infer an estimated clone size based on Mutation Frequency (MF), which is the proportion of reads containing the mutated allele compared with the total number of reads, adjusted for chromosome copy number. Condensed results are shown in Table 2.

**Table 1:** Clinical features of the AML patients enrolled in this study

Patient ID	Gender	Age atdiagnosis(years)	FAB subtype	WBC atdiagnosis(10^9^/L)	BM blastsat diagnosis(%)	WBC at relapse (10^9^/L)	BM blastsat relapse(%)	Extramedullary involvement	Time torelapse(months)	HSCT (type)
^*^ **AML#1**	M	3.3	M2	12.01	70	3.21	60	NO	24	YES ^1^ (AUTO)
**AML#2**	M	14.5	M5	114.04	92	53.01	80	NO	14	YES ^2^ (MUD)
**AML#3**	F	7.4	M4	4.02	53	1.7	70	NO	11	YES ^2^ (MUD)
^*^ **AML#4**	M	12.6	M1	14	80	6.9	24	YES (Tonsils)	18	YES ^2^ (AUTO)

* patient alive and in CR;

1 HSCT in 1^st^ CR;

2 HSCT in 2^nd^ CR;

HSCT, hematopoietic stem cell transplantation; AUTO, autologous; MFD, matched family donor; MUD, matched unrelated donor; WBC, white blood cells.

**Table 2:** Results of targeted deep sequencing of candidate driver mutations

Gene	Patient	Chr	Start	Ref	Alt	cDNA	Protein	MF at diagnosis	Inferred clone size at diagnosis^*^	MF at remission	MF at relapse	Inferred clone size at relapse^*^
TYK2	AML#1	19	10467264	A	T	c.T2597A	p.L866H	43,0%	80-90%	0,40%	14,9%	30%
RREB1	AML#2	6	7231420	G	A	c.G3088A	p.V1030M	57,6%	100%	ND	53,1%	100%
PSIP1	AML#2	9	15469966	G	T	c.C1003A	p.P335T	ND	0%	ND	43,9%	40-50%
ASXL3	AML#2	18	31324221	C	T	c.C4409T	p.P1470L	0,3%	<1%	ND	29,7%	60%
WT1	AML#2	11	32438075	C	G	c.G962C	p.G321A	ND	0%	ND	40,0%	40%
CEBPA	AML#2	19	33792384	-	CTG	c.937_938 insCAG	p.K313 delinsQK	88,6%	100%	0,08%	80,8%	100%
SETD2	AML#2	3	47098968	-	GGTG	c.6306_6307 insCACC	p.P2102fs	32,5%	60-70%	0,07%	31,7%	60-70%
WISP1	AML#2	8	134239889	G	A	c.G1040A	p.R347K	ND	0%	ND	34,0%	60-70%
FLT3	AML#3	13	28592640	A	T	c.T2505A	p.D835E	3,4%	<10%	ND	13,3%	25-30%
WT1	AML#3	11	32417913	-	GTACAAGA	c.1139_1140 insTCTTGTAC	p.R380fs	13,9%	25-30%	ND	4,2%	<10%
SALL1	AML#3	16	51174616	A	G	c.T1517C	p.I506T	ND	0%	ND	28,6%	50-60%
UBE2D3	AML#3	4	103722704	T	C	c.A211G	p.T71A	ND	0%	ND	27,7%	50-60%
PTPN11	AML#3	12	112888199	C	T	c.C215T	p.A72V	ND	0%	ND	31,9%	60-70%
TEK	AML#4	9	27212867	G	A	c.G2849A	p.R950Q	ND	0%	ND	21,0%	40-50%
WT1	AML#4	11	32417907	-	CCGA	c.1145_1146 insTCGG	p.A382fs	27,6%	55%	ND	ND	0%

Chr: chromosome; Ref: reference; Alt: alteration; MF: mutation frequency; ND: not detected;

* corrected for copy number variations.

### BiCEBPα mutations are highly persistent contrary to WT1 mutations

**Figure 2:**
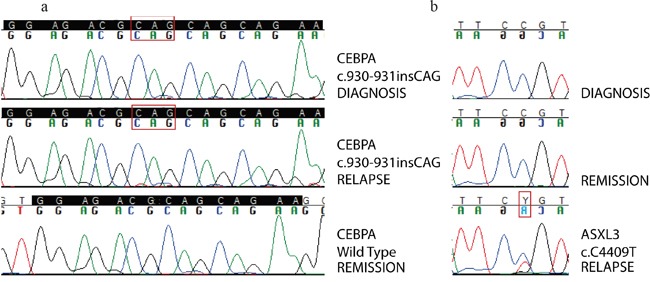
Sanger sequencing validation of somatic mutations **Panel 2a:** Sanger validation of biCEBPα mutation in patient AML#2. Homozygous CAG in-frame insertion is detected in the whole tumor population both at diagnosis and at relapse, while is not detected in remission sample used as germinal counterpart. High coverage targeted deep sequencing was actually able to detect a very small clone (< 0,1%) carrying this mutation persisting at remission. **Panel 2b:** Sanger validation of ASXL3 mutation in patient AML#2. Point mutation c.C4409T is detected only at relapse. Backtracking of this mutation through high coverage targeted deep sequencing was able to detect a minor subclone carrying this mutation even at diagnosis.

We detected a highly penetrant biallelic mutation of CEBPα (biCEBPα). In patient AML#2, both WES and targeted deep sequencing showed a homozygous non-frameshift insertion (c.937_938insCAG, p.K313delinsQK) of CEBPα, involving the bZIP domain, in the majority of tumor-cell population, as revealed by MF>80%, both at diagnosis and at relapse. Validation by Sanger sequencing is shown in Figure 2a. On the contrary, WT1 mutations appeared highly unstable. WT1 codes for a transcriptional factor recurrently mutated in AML [13], but with a still unclear role in leukemia development [14]. The following WT1 mutations were found: frameshift insertion c.1145_1146insTCGG (p.A382fs) in AML#4 (detected only at diagnosis with a MF of 27.6%) and frameshift insertion c.1139_1140insTCTTGTAC (p.R380fs) in AML#3 [detected both at diagnosis (MF 13.9%) and at relapse (MF 4,2%)]. These insertions involve the hotspot mutational area of exon 7, associated with AML, resulting in loss of the zinc-finger DNA-binding domain of the protein [13]. Moreover, SNV c.G962C (p.P1470L) was detected only in the relapse sample of AML#2 with a MF of 40%, associated with loss of a copy of the wild type allele at relapse. The mutation involves exon 5, which is not a common site of mutations in AML [13].

### Detection of genomic mechanisms underlying relapse in AML

Sequencing of primary tumor–relapse pairs allowed us identifying specific mutation patterns associated with relapse. In patient AML#3, we detected the acquisition of an activating mutation (p.A72V) of PTPN11 gained at relapse (MF 31.9%). In the same patient, targeted deep sequencing revealed a minor subclone carrying a FLT3-TKD mutation (p.D835E) already present at diagnosis (MF 3.4%), although below the sensitivity of WES, and increasing at relapse (MF 13.3%). Given the discordant increase of MF of PTPN11 and FLT3-TKD, it is reasonable to hypothesize that each characterized a different clone.

In patient AML#1, we detected a point mutation (c.T2597A, p.L866H) involving the pseudokinase domain of TYK2 both in the diagnosis (MF of 43%) and relapsed (MF of 14.9%) samples. The pseudokinase domain of this protein plays a role in the inhibition of the kinase domain, and many cancer-associated mutations described lie in or near the interface between these two domains, resulting in increased kinase activity *in vitro* [15] [16]. To the best of our knowledge, the mutation we identified has not been previously reported, but, considering its involvement of the pseudokinase domain, we can speculate it causes an increased kinase activity.

In patient AML#2, we detected a frameshift insertion (c.6306_6307insCACC, p.P2102fs) of gene SETD2 in a considerable fraction of the tumor population both at diagnosis (MF 32.5%) and at relapse (MF 31.7%). SETD2 is a methyltransferase responsible for H3K36 trimethylation (H3K36me3), which, in turn, is responsible for the recruitment of mismatch repair (MMR) machinery. This mutation involves the Set2-Rpb1 interacting (SRI) domain at the C-terminal segment, which interacts with the phosphor-C-terminal repeat domain (PCTD) of elongating RNA polymerase II and leads to the recruitment of SETD2 to its target genes [17]. Disruption of this domain due to either missense or truncating mutations has been previously reported as causing loss of function of the methyltransferase activity [18].

### Detection of a novel ASXL3 mutation as a late event

In patient AML#2, point mutation c.C4409T (p.P1470L) of ASXL3 was detected at relapse (MF of 29.7%) and backtracked in a very small subclone already present in the primary sample (MF of 0.3%). This mutation was predicted to be deleterious at protein level. Sanger sequencing validation is shown in Figure 2b. This represents one of the few examples of ASXL3 mutations described in AML [19] [11], as opposed to mutations of ASXL1 and ASXL2, the other two members of the Additional Sex combs (Asx)-Like family, which appear to be quite commonly mutated in AML [19].

## DISCUSSION

Given the remarkable frequency of relapse in childhood AML and the poor prognosis associated with disease recurrence [5] [6], deeper information on the mechanisms responsible for relapse is desirable to improve response to treatment and survival. An important point in understanding leukemia genomics is to identify leukemia-initiating mutations, i.e. the so called “primary events” that result in leukemic transformation. This notion will lead us to target the disease at its origin. Despite great progress in the last decades, in the majority of cases this still remains an unsolved question. However, an equally important task is to understand how leukemia evolves once this transformation has happened. A better knowledge of this process will allow us to refine disease monitoring and to choose optimal therapies both in terms of molecular targets and schedule.

The term “clonal evolution” refers to tumor progression through stepwise acquisition of new mutations providing genetic diversity within a cell lineage. The dynamics of this process depend on the interaction between the specific effect of new mutations and micro-environmental conditions, such as resource limitations and chemotherapy. This results in selection and expansion of more fit subclones, together with eradication or self-extinction of less fit subclones [20]. Our results, graphically plotted in Figure 1, clearly show clonal evolution from diagnosis to relapse, further confirming the very recent findings of Farrar et al [10]. In fact, while the genetic alterations shared between the primary-tumor and the relapse samples prove a common origin from an ancestral clone, the evidence of diagnosis-specific and relapse-specific mutations strongly supports a branching model of clonal progression. “Primary events” responsible for the origin of AML are highly penetrant in the tumor population and stable during the course of disease, that is continuously present in one patient's leukemia from diagnosis to relapse. On the other end of the spectrum, “secondary events” occur later and only in a fraction of cells, conferring a greater amount of complexity to the genomic profile of the disease [21]. In order to clarify if clones giving rise to relapse were already present at diagnosis but below the threshold of detection of WES, or they arose later in the course of the disease, we performed targeted deep sequencing of putative driver mutations. Interestingly, targeted deep sequencing was also able to detect persistence of some leukemia-related mutations (biCEBPα, TYK2 and SETD2) during relapse at a very low MF (0.08%, 0.4% and 0.07% respectively), further supporting the potential application of Next Generation Sequencing techniques in minimal residual disease monitoring [22] [23] [24]. On the contrary, the great instability of WT1 mutations, both in terms of loss and acquisition, concordant with previous reports [10] [13] [25] [26], suggests caution in adopting WT1 monitoring-based techniques. According to the model described above, biCEBPα mutation, defined as disruption of both CEBPα alleles [27], was detected in the vast majority of the tumor-cell population of one of our patients, both at diagnosis and at relapse. This finding mirrors the pathogenetic role of the event: CEBPα, in fact, appears to play multiple roles in normal hematopoiesis, both in regulating differentiation and cell proliferation, and its disruption alone results in the accumulation of blasts [28]. Thus, the stability of biCEBPα supports its role in defining a distinct molecular and clinical subtype of AML [29], as reported in the 2016 WHO classification [30].

**Figure 3:**
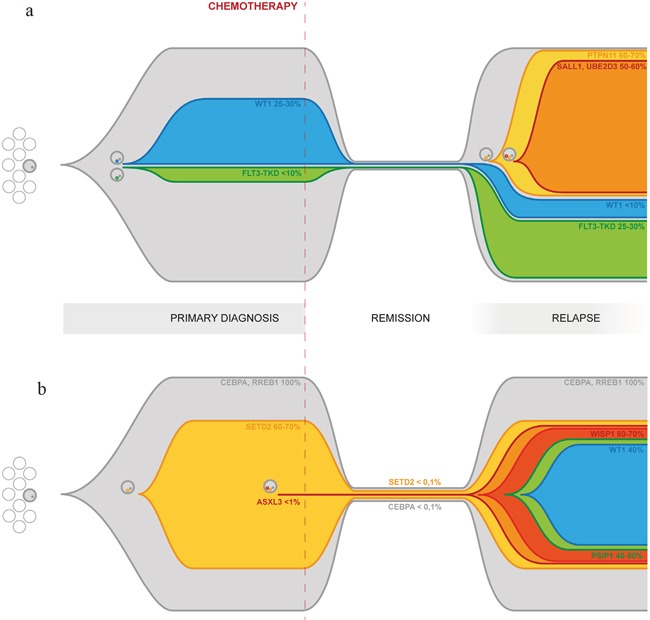
Graphical representation of clonal evolution from primary diagnosis to relapse based on targeted deep sequencing of driver mutations **Panel 3a:** Clonal evolution in patient AML#3. The primary tumor differentiates into subclones through the acquisition of new somatic mutations, including WT1 and FLT3-TKD. Those clones survive chemotherapy and contribute to relapse. Later acquisition of additional mutations, such as PTPN11, SALL1 and UBE2D3, further increases clonal heterogeneity and confers a higher degree of complexity to the disease. Reported percentages refer to the estimated size population of each clone inferred from the MF calculated on targeted deep sequencing data for each mutation and adjusted for CN. **Panel 3b:** Clonal evolution in patient AML#2. The entire tumor population both in the primary and in the relapse samples carries a biCEBPα and a RREB1 mutation. The inactivation of SETD2 in a substantial fraction of the cells is associated with the acquisition of a mutator phenotype causing differentiation into multiple minor subclones through the acquisition of additional somatic mutations, increasing the plasticity and adaptability of the leukemia. High coverage targeted deep sequencing was able to detect persistence of biCEBPAα and SETD2 mutation during remission. Reported percentages refer to the estimated size population of each clone inferred from the MF calculated on targeted deep sequencing data for each mutation and adjusted for CN.

The aim of our work was to uncover mutational patterns underlying relapse. The first pattern we report is related to an increased proliferation signal giving significant advantage to a specific clone. This model is well depicted in Figure 3a, tracing the dynamics of clonal evolution in patient AML#3. A small FLT3-TKD-mutated subclone (<10%) of the primary leukemia survived chemotherapy and underwent expansion at relapse (25-30%). An additional clone, characterized by the acquisition of a PTPN11 mutation, appeared later, but overcame other clones becoming the predominant one at relapse (60-70%). It is well known that both these kinds of events lead to increased cell proliferation and/or survival [31] [32]. Not only the interaction of the mutation effect with the environment, but also the mutual competition of various subclones and the proliferative kinetics of each one within the tumor, turn out to be fundamentals of clonal evolution. Although these late events may not be necessary for leukemogenesis *per se*, they clearly play an important role in disease progression by conferring a specific proliferative advantage. This is particularly relevant considering that these mutations could potentially be the target for tailored therapy. Moreover, FLT3/TKD and ITD being subclonal mutations is one of the plausible explanations of unsatisfying results of FLT3 inhibitors, along with many others concerning inadequate *in vivo* inhibition of the target, development of secondary pharmacokinetic or pharmacodynamic resistance, and influence of FLT3-mutant allelic burden. Hence, accurate molecular characterization of the disease also at relapse can guide the choice of optimal therapies, even targeting the various subclones within the bulk tumor by using multiple agents simultaneously.

A fitness advantage can also be expressed as an abnormal resistance to apoptosis causing clonal survival to therapy. This feature was found in patient AML#1, where a clone carrying a deleterious point mutation of TYK2, a member of the janus tyrosine kinases (JAK) family, contributed to relapse. These cytoplasmic kinases mediate intracellular activation of a variety of pathways affecting cellular growth, differentiation and survival [33]. Hyperactivation of TYK2 pathway, either through mutations or other mechanisms, has been shown to contribute to aberrant cell survival through upregulation of the anti-apoptotic protein BCL2 in several T-ALL-cell lines [34]. There is also growing evidence for a role of the anti-apoptotic members of BCL2 family in promoting therapy resistance and survival both in AML and myelodysplastic syndromes [35] [36] [37]. In the same way, a TYK2-activating mutation likely promoted clonal survival to therapy in our patient. This clone represented about a third of the whole blast population at relapse; thus, unknown events must have promoted survival of other clones. Nonetheless, a single relapse sample is a one-shot picture where, as described above, the relative proportion of various clones is strongly related to the kinetics of dividing cells. In the long run instead, persistence of a reservoir of cells ‘throughout’ therapy can also be related to a more stable or even quiescent state.

A third and unexpected pattern underlying clonal evolution is the acquisition of a mutator phenotype marked by accumulation of a large number of mostly subclonal mutations due to failure of DNA repair. This has already been described in cases of relapsed ALL [38] [39], while is a surprisingly new feature in AML, which is traditionally known as characterized by a very low number of somatic mutations and absence of genomic instability [11]. Indeed, in patient AML#2, both in the diagnosis and the relapse samples, we detected a much higher burden of mutations than in the other three analyzed. This mutator phenotype is associated with a SETD2 truncating mutation, able to disrupt its function in the recruitment of the MMR machinery [40]. Besides being found in clear cell renal carcinoma [41] [42], SETD2 mutations have recently been identified by Zhu et al [18] in 6.2% out of 241 patients with acute leukemia (both AML and ALL). SETD2 mutations have also been described as gained during relapse in childhood ALL [43]. Our results further support the idea of a role of SETD2 mutations in AML, particularly contributing to clonal selection and survival. In fact, the accumulation of additional mutations, dramatically increasing the plasticity and adaptability of the leukemia cells, leads in the end to a higher chance of escaping therapy. A graphical representation of clonal evolution in patient AML#2 from diagnosis to relapse shown in Figure 3b.

Finally, we also identified a novel point mutation of ASXL3. It was detected in a very small subclone (<1%) in the primary sample, but underwent expansion (60%) at relapse. While mutations of the other two members of Additional Sex combs (Asx)-Like family, ASXL1 and ASXL2, are recurrently mutated in AML, mutations of ASXL3 have been described only twice in the setting of AML so far [11] [19]. Moreover, while ASXL1 mutations, according to their role in modulating gene expression through epigenetic regulation, are regarded as “landscaping” events early initiating leukemogenesis at a pre-leukemic stage [44] [45], the ASXL3 mutation we identified appears to be a late event.

In summary, we ultimately uncovered the polyclonal structure of pediatric AML, revealing a global shifting in the mutational spectrum from diagnosis to relapse. Our results further confirm the recent evidence [10] of clonal evolution in childhood AML and highlight a remarkable and previously unknown genomic complexity of the disease. Possible patterns of clonal evolution are various and heterogenous in each single patient, further supporting the need for individualized diagnostic and therapeutic strategies. Moreover, monitoring the dominant clone detected at diagnosis may not be a reliable marker of impending relapse, while the detection and track of emerging subclones may be more informative. With great technological improvements of sequencing platforms in the last and future years, both in terms of time needed to perform the analysis and costs, we may predict that a similar approach will soon be available to clinical use. These results provide greater knowledge of the clonal architecture underlying relapse. More in depth, considering the different types of genes involved, we proved the idea [46] that once the leukemic transformation has occurred, clonal evolution results from a complex interplay of ‘driver’ lesions, such as mutations providing a proliferative advantage (i.e. FLT3 or PTPN11 activating mutations) or an increased survival (i.e TYK2 mutations), as well as ‘deleterious’ lesions, causing self-extinction, and ‘passenger’ lesions. We also found a ‘mutator’ lesion (i.e. SETD2 inactivation) as a previously undescribed way of increasing the rate of other genetic changes therefore promoting the perpetuating of the disease.

## MATERIALS AND METHODS

### Patient samples

Patient samples analyzed were collected at the time of diagnosis, first complete remission and relapse, respectively, from 4 children with *de novo* AML other than promyelocytic leukemia, enrolled in the Associazione Italiana Ematologia Oncologia Pediatrica (AIEOP) 2002/01 Study [3] after obtaining written informed consent from the parents according to the Declaration of Helsinki. FAB morphological diagnosis and immunophenotypic analysis was centrally reviewed at the laboratory of Pediatric Haematology of the University Hospital in Padova. Chromosome analysis was performed on bone marrow (BM) aspirates using standard laboratory procedures. Karyotypes were reported according to the International System for Human Cytogenetic Nomenclature (ISCN 2005). For fluorescence *in-situ* hybridization (FISH), an MLL locus specific (LSI) dual colour probe for 11q23 (Abbott-Vysis, Downers Grove, IL) was employed. Clinical features of the patients are reported in Table 1.

### Whole-exome sequencing and bioinformatics analyses

Total DNA was extracted from BM leukemia/mononucleated cells of the 4 AML patients by QIAamp DNA Mini kit (Qiagen) and exome library preparation was performed by Nextera Rapid Capture Enrichement kit (Illumina, San Diego, CA) according to the manufacturer's recommendations. Bridge amplification was conducted through cBot cluster amplification system/TruSeq PE Cluster Kit v3-cBot-HS (Illumina). Sequencing by synthesis was performed on HiScanSQ sequencer (Illumina) at 100 bp in paired-end mode. After adapter and quality trimming, implemented by AdapterRemoval algorithm [47], reads were aligned with Burrows-Wheeler Aligner [48] to the reference human genome hg19/GRCh37. *Genome Analysis Toolkit* (GATK) (*Haplotype Caller*) and *MuTect* packages were used to detect *Ins/Dels* and SNVs respectively from exome-seq data [49] [50]. Variants present in dbSNP database (http://www.ncbi.nlm.nih.gov/project/SNP), 138 edition, and in 1000 *Genomes* database (http://wwww.1000genomes.org), February 2012 edition with frequency greater than 1% were excluded. Synonym SNVs were excluded, thus resulting in evaluating only non-synonymous SNVs, nonsense SNVs, SNVs at splicing sites and *Ins/Dels*, both frameshift and non-frameshift. In further analysis, only high-quality somatic mutations were considered, according to their absence in the control sample obtained at time of complete remission from the corresponding patient (for detailed criteria refer to Supplementary Information). The effect of mutations at the protein level was predicted with the computational tools SIFT and PROVEAN [51]. Copy number alterations were detected by Control-FREEC Software [52] on WES data, comparing primary and relapse samples to remission sample of corresponding patient.

### Targeted deep sequencing of candidate driver mutations on MiSeq

In order to clarify molecular mechanisms underlying disease relapse, among mutations detected by WES, we further selected candidate driver mutations for targeted deep sequencing, based on a comprehensive analysis of their recurrence in AML or on a possible pathogenetic role, according to literature. DNA library preparation was performed with Nextera XT DNA Prep kit (Illumina). Amplicons of the corresponding regions were prepared through PCR reaction with AmpliTaq FastStart kit (Applied Biosystem). The full list of primers used at this stage is reported in Supplementary Table S2. Deep sequencing was performed on MiSeq System (Illumina) according to the manufacturer's recommendations. The complete output of targeted deep sequencing is reported in Supplementary Table S3. For a detailed description of output data analysis refer to Supplementary Information.

## SUPPLEMENTARY MATERIALS TABLES



## References

[1] Zwaan CM, Kolb EA, Reinhardt D, Abrahamsson J, Adachi S, Aplenc R, De Bont ESJM, De Moerloose B, Dworzak M, Gibson BES, Hasle H, Leverger G, Locatelli F (2015). Collaborative efforts driving progress in pediatric acute myeloid leukemia. J. Clin. Oncol.

[2] Gibson BES, Webb DKH, Howman AJ, De Graaf SSN, Harrison CJ, Wheatley K (2011). Results of a randomized trial in children with Acute Myeloid Leukaemia: medical research council AML12 trial. Br. J. Haematol.

[3] Pession A, Masetti R, Rizzari C, Putti MC, Casale F, Fagioli F, Luciani M, Lo Nigro L, Menna G, Micalizzi C, Santoro N, Testi AM, Zecca M (2013). Results of the AIEOP AML 2002/01 multicenter, prospective trial for treatment of children with acute myeloid leukemia. Blood.

[4] Creutzig U, van den Heuvel-Eibrink MM, Gibson B, Dworzak MN, Adachi S, de Bont E, Harbott J, Hasle H, Johnston D, Kinoshita A, Lehrnbecher T, Leverger G, Mejstrikova E (2012). Diagnosis and management of acute myeloid leukemia in children and adolescents: recommendations from an international expert panel. Blood.

[5] Sander A, Zimmermann M, Dworzak M, Fleischhack G, von Neuhoff C, Reinhardt D, Kaspers GJL, Creutzig U (2010). Consequent and intensified relapse therapy improved survival in pediatric AML: results of relapse treatment in 379 patients of three consecutive AML-BFM trials. Leukemia.

[6] Kaspers GJL, Zimmermann M, Reinhardt D, Gibson BES, Tamminga RYJ, Aleinikova O, Armendariz H, Dworzak M, Ha SY, Hasle H, Hovi L, Maschan A, Bertrand Y (2013). Improved outcome in pediatric relapsed acute myeloid leukemia: results of a randomized trial on liposomal daunorubicin by the international BFM study group. J. Clin. Oncol.

[7] Gorman MF, Ji L, Ko RH, Barnette P, Bostrom B, Hutchinson Raymond, Raetz E, Seibel NL, Twist CJ, Eckroth E, Sposto R, Gaynon PS, Loh ML (2010). Outcome for children treated for relapsed or refractory Acute Myelogenous Leukemia (rAML): a therapeutic advances in childhood leukemia (TACL) consortium study. J. Clin. Oncol.

[8] de Rooij J, Zwaan C, van den Heuvel-Eibrink M (2015). Pediatric AML: from biology to clinical management. J. Clin. Med.

[9] Ding L, Ley TJ, Larson DE, Miller C, Koboldt DC, Welch JS, Ritchey JK, Young M, Lamprecht T, McLellan MD, McMichael JF, Wallis JW, Lu C (2012). Clonal evolution in relapsed acute myeloid leukaemia revealed by whole-genome sequencing. Nature.

[10] Farrar JE, Schuback HL, Ries RE, Wai D, Hampton OA, Trevino LR, Alonzo TA, Guidry Auvil JM, Davidsen TM, Gesuwan P, Hermida L, Muzny DM, Dewal N (2016). Genomic profiling of pediatric acute myeloid leukemia reveals a changing mutational landscape from disease diagnosis to relapse. Cancer Res.

[11] Cancer Genome Atlas Research Network (2013). Genomic and epigenomic landscapes of adult de novo acute myeloid leukemia. N. Engl. J. Med.

[12] Walter MJ, Payton JE, Ries RE, Shannon WD, Deshmukh H, Zhao Y, Baty J, Heath S, Westervelt P, Watson MA, Tomasson MH, Nagarajan R, O’Gara BP (2009). Acquired copy number alterations in adult acute myeloid leukemia genomes. Proc. Natl. Acad. Sci.

[13] Hollink I, Van den Heuvel-Eibrink MM, Zimmermann M, Balgobind B, Arentsen-Peters STCJM, Alders M, Willasch A, Kaspers GJL, Trka J, Baruchel A, de Graaf SSN, Creutzig U, Pieters R (2009). Clinical relevance of Wilms tumor 1 gene mutations in childhood acute myeloid leukemia. Blood.

[14] Ho PA, Zeng R, Alonzo TA, Gerbing RB, Miller KL, Pollard JA, Stirewalt DL, Heerema NA, Raimondi SC, Hirsch B, Franklin JL, Lange B, Meshinchi S (2010). Prevalence and prognostic implications of WT1 mutations in pediatric acute myeloid leukemia (AML): a report from the Children's Oncology Group. Blood.

[15] Min X, Ungureanu D, Maxwell S, Hammarén H, Thibault S, Hillert E-K, Ayres M, Greenfield B, Eksterowicz J, Gabel C, Walker N, Silvennoinen O, Wang Z (2015). Structural and functional characterization of the JH2 pseudokinase domain of JAK Family Tyrosine Kinase 2 (TYK2). J. Biol. Chem.

[16] Lupardus PJ, Ultsch M, Wallweber H, Bir Kohli P, Johnson AR, Eigenbrot C (2014). Structure of the pseudokinase-kinase domains from protein kinase TYK2 reveals a mechanism for Janus kinase (JAK) autoinhibition. Proc. Natl. Acad. Sci. U. S. A.

[17] Li M, Phatnani HP, Guan Z, Sage H, Greenleaf AL, Zhou P (2005). Solution structure of the Set2-Rpb1 interacting domain of human Set2 and its interaction with the hyperphosphorylated C-terminal domain of Rpb1. Proc. Natl. Acad. Sci. U. S. A.

[18] Zhu X, He F, Zeng H, Ling S, Chen A, Wang Y, Yan X, Wei W, Pang Y, Cheng H, Hua C, Zhang Y, Yang X (2014). Identification of functional cooperative mutations of SETD2 in human acute leukemia. Nat Genet.

[19] Duployez N, Micol JB, Boissel N, Petit A, Geffroy S, Bucci M, Lapillonne H, Renneville A, Leverger G, Ifrah N, Dombret H, Abdel-Wahab O, Jourdan E (2016). Unlike ASXL1 and ASXL2 mutations, ASXL3 mutations are rare events in acute myeloid leukemia with t(8;21). Leuk. Lymphoma.

[20] Jan M, Majeti R (2013). Clonal evolution of acute leukemia genomes. Oncogene.

[21] Grove CS, Vassiliou GS (2014). Acute myeloid leukaemia: a paradigm for the clonal evolution of cancer? Dis. Model. Mech.

[22] Bibault J, Figeac M, Hélevaut N, Quief S, Sebda S, Renneville A, Rousselot P, Gruson B, Dombret H (2015). Next-generation sequencing of FLT3 internal tandem duplications for minimal residual disease monitoring in acute myeloid leukemia. Oncotarget.

[23] Kohlmann A, Nadarajah N, Alpermann T, Grossmann V, Schindela S, Dicker F, Roller A, Kern W, Haferlach C, Schnittger S, Haferlach T (2014). Monitoring of residual disease by next-generation deep-sequencing of RUNX1 mutations can identify acute myeloid leukemia patients with resistant disease. Leukemia.

[24] Klco JM, Miller CA, Griffith M, Petti A, Spencer DH, Ketkar-kulkarni S, Wartman LD, Christopher M, Lamprecht TL, Helton NM, Duncavage EJ, Payton JE, Baty J (2015). Association between mutation clearance after induction therapy and outcomes in Acute Myeloid Leukemia. JAMA.

[25] Hou HA, Huang TC, Lin LI, Liu CY, Chen CY, Chou WC, Tang JL, Tseng MH, Huang CF, Chiang YC, Lee FY, Liu MC, Yao M (2010). WT1 mutation in 470 adult patients with acute myeloid leukemia: stability during disease evolution and implication of its incorporation into a survival scoring system. Blood.

[26] Nyvold CG, Stentoft J, Braendstrup K, Melsvik D, Moestrup SK, Juhl-Christensen C, Hasle H, Hokland P (2006). Wilms’ tumor 1 mutation accumulated during therapy in acute myeloid leukemia: biological and clinical implications. Leukemia.

[27] Ho PA, Alonzo TA, Gerbing RB, Pollard J, Stirewalt DL, Hurwitz C, Heerema NA, Hirsch B, Raimondi SC, Lange B, Franklin JL, Radich JP, Meshinchi S (2009). Prevalence and prognostic implications of CEBPA mutations in pediatric acute myeloid leukemia (AML): a report from the Children's Oncology Group. Blood.

[28] Mueller BU, Pabst T (2006). C/EBPalpha and the pathophysiology of acute myeloid leukemia. Curr. Opin. Hematol..

[29] Wouters BJ, Löwenberg B, Erpelinck-Verschueren CAJ, Van Putten WLJ, Valk PJM, Delwel R (2009). Double CEBPA mutations, but not single CEBPA mutations, define a subgroup of acute myeloid leukemia with a distinctive gene expression profile that is uniquely associated with a favorable outcome. Blood.

[30] Arber DA, Orazi A, Hasserjian R, Thiele J, Borowitz MJ, Le Beau MM, Bloomfield CD, Cazzola M, Vardiman JW (2016). The 2016 revision to the World Health Organization (WHO) classification of myeloid neoplasms and acute leukemia. Blood.

[31] Tartaglia M, Martinelli S, Iavarone I, Cazzaniga G, Spinelli M, Giarin E, Petrangeli V, Carta C, Masetti R, Aricò M, Locatelli F, Basso G, Sorcini M (2005). Somatic PTPN11 mutations in childhood acute myeloid leukaemia. Br. J. Haematol.

[32] Liang D-C, Shih L-Y, Hung I-J, Yang C-P, Chen S-H, Jaing T-H, Liu H-C, Wang L-Y, Chang W-H (2003). FLT3-TKD mutation in childhood acute myeloid leukemia. Leukemia.

[33] Meyer S, Levine R (2014). Molecular pathways: molecular basis for sensitivity and resistance to JAK kinase inhibitors. Clin Cancer Res.

[34] Sanda T, Tyner JW, Gutierrez A, Ngo VN, Glover J, Chang BH, Yost A, Ma W, Fleischman AG, Zhou W, Yang Y, Kleppe M, Ahn Y (2013). TYK2-STAT1-BCL2 Pathway Dependence in T-Cell Acute Lymphoblastic Leukemia. Cancer Discov.

[35] Chan SM, Thomas D, Corces-Zimmerman MR, Xavy S, Rastogi S, Hong W-J, Zhao F, Medeiros BC, Tyvoll DA, Majeti R (2015). Isocitrate dehydrogenase 1 and 2 mutations induce BCL-2 dependence in acute myeloid leukemia. Nat. Med.

[36] Cluzeau T, Robert G, Mounier N, Karsenti JM, Dufies M, Puissant A, Jacquel A, Renneville A, Preudhomme C, Cassuto J-P, Raynaud S, Luciano F, Auberger P (2012). BCL2L10 is a predictive factor for resistance to azacitidine in MDS and AML patients. Oncotarget.

[37] Zhou W, Xu J, Gelston E, Wu X, Zou Z, Wang B, Zeng Y, Wang H, Liu A, Xu L, Liu Q (2015). Inhibition of Bcl-xL overcomes polyploidy resistance and leads to apoptotic cell death in acute myeloid leukemia cells. Oncotarget.

[38] Kunz JB, Rausch T, Bandapalli OR, Eilers J, Pechanska P, Schuessele S, Assenov Y, Stütz AM, Kirschner-Schwabe R, Hof J, Eckert C, von Stackelberg A, Schrappe M (2015). Pediatric T-lymphoblastic leukemia evolves into relapse by clonal selection, acquisition of mutations and promoter hypomethylation. Haematologica.

[39] Ma X, Edmonson M, Yergeau D, Muzny DM, Hampton OA, Rusch M, Song G, Easton J, Harvey RC, Wheeler DA, Ma J, Doddapaneni HV, Vadodaria B (2015). Rise and fall of subclones from diagnosis to relapse in pediatric B-acute lymphoblastic leukaemia. Nat. Commun.

[40] Li F, Mao G, Tong D, Huang J, Gu L, Yang W, Li G (2013). The histone mark H3K36me3 regulates human DNA Mismatch Repair through its interaction with MutSα. Cell.

[41] Xiang W, He J, Huang C, Chen L, Tao D, Wu X (2015). miR-106b-5p targets tumor suppressor gene SETD2 to inactive its function in clear cell renal cell carcinoma. Oncotarget.

[42] Tiedemann RL, Hlady RA, Hanavan PD, Lake DF, Tibes R, Lee J, Choi J, Ho TH, Robertson KD (2015). Dynamic reprogramming of DNA methylation in SETD2-deregulated renal cell carcinoma. Oncotarget.

[43] Mar BG, Bullinger LB, Mclean KM, Grauman P V, Harris MH, Stevenson K, Neuberg DS, Sinha AU, Sallan SE, Silverman LB, Kung AL, Lo Nigro L, Ebert BL (2014). Mutations in epigenetic regulators including SETD2 are gained during relapse in pediatric acute lymphoblastic leukemia. Nat Commun.

[44] Corces-Zimmerman MR, Hong W-J, Weissman IL, Medeiros BC, Majeti R (2014). Preleukemic mutations in human acute myeloid leukemia affect epigenetic regulators and persist in remission. Proc. Natl. Acad. Sci. U. S. A.

[45] Majeti R (2014). Clonal evolution of preleukemic hematopoietic stem cells precedes human acute myeloid leukemia. Best Pract. Res. Clin. Haematol.

[46] Greaves M, Maley C (2012). Clonal evolution in cancer. Nature.

[47] Lindgreen S (2012). AdapterRemoval: easy cleaning of next-generation sequencing reads. BMC Res Notes.

[48] Li H, Durbin R (2010). Fast and accurate long-read alignment with Burrows-Wheeler transform. Bioinformatics.

[49] McKenna A, Hanna M, Banks E, Sivachenko A, Cibulskis K, Kernytsky A, Garimella K, Altshuler D, Gabriel S, Daly M, DePristo MA (2010). The Genome Analysis Toolkit: a MapReduce framework for analyzing next-generation DNA sequencing data. Genome Res.

[50] Cibulskis K, Lawrence MS, Carter SL, Sivachenko A, Jaffe D, Sougnez C, Gabriel S, Meyerson M, Lander ES, Getz G (2013). Sensitive detection of somatic point mutations in impure and heterogeneous cancer samples. Nat Biotechnol.

[51] Choi Y, Sims GE, Murphy S, Miller JR, Chan AP (2012). Predicting the functional effect of amino acid substitutions and indels. PLoS One.

[52] Boeva V, Popova T, Bleakley K, Chiche P, Cappo J, Schleiermacher G, Janoueix-Lerosey I, Delattre O, Barillot E (2012). Control-FREEC: a tool for assessing copy number and allelic content using next-generation sequencing data. Bioinformatics.

