# Supplementary material for: Genomic complexity and dynamics of clonal evolution in childhood acute myeloid leukemia studied with whole-exome sequencing
Source: Oncotarget. 2016 Jul 22;7(35):56746–57. doi: 10.18632/oncotarget.10778 (PMC5302950; doi:10.18632/oncotarget.10778)
Supplement: Supplementary file 1 [file oncotarget-07-56746-s001.pdf]

Genomic complexity and dynamics of clonal evolution in childhood acute myeloid leukemia studied with whole-exome sequencing

SUPPLEMENTARY DATA

Bioinformatics analysis of whole-exome sequencing data

Whole-exome sequencing(WES) of primary tumor–relapse pairs and matched remission samples from 4 childhood AML patients was conducted by HiScanSQ sequencer (Illumina, San Diego, CA) and resulted in 9,34X10^8 reads, yielding >95% diploid exome coverage referring to Nextera Rapid Capture target region (37 Mb). Average haploid coverage of targeted regions (10X) for each sample was between 51X and 75X. 4 340 023 SNVs and 406 207 *Ins/Dels* (Insertions/Deletions) were found among the 12 samples, 4 172 533 (88%) of which were excluded as polymorphic variants. Synonymous SNVs were also rejected; 87% of the remaining variants were discarded since they were considered germinal variants or indeterminate. Thus only somatic variants were further examined, according to the following criteria:

-Somatic variants: Total Remission Coverage >=10 and <15 with Remission Variant Coverage =0; or

Total Remission Coverage >=15 and<30 with Remission Variant Coverage<=1; or

Total Remission Coverage >=30 with Remission Variant Coverage<=2;

-Indeterminate variants: Total Remission Coverage <10

-Germline variant: neither somatic nor indeterminate

Finally, only high quality somatic mutations were evaluated according to the following criteria in at least either primary sample or relapse sample:

-Total Coverage >=15

-Variant Coverage > =5

-PASS or KEEP quality output from GATK

-Manual revision through Integrative Genomic Viewer software (http://www.broadinstitue.org/software/igv/home)

The list of all novel somatic SNVs and *Ins/Dels* for each patient is provided in Supplementary Table 1. 1-1.4, resulting as following: 6 mutations in AML#1, 41 in AML#2, 11 in AML#3, 7 in AML#4. Effect on protein function was predicted using bioinformatics tools (SIFT and PROVEAN).

Targeted deep sequencing on MiSeq System (Illumina) of candidate somatic mutations

Targeted deep sequencing of candidate driver mutations was performed on MiSeq System (Illumina). The full list of primers used at this stage is reported in Supplementary Table 2. Average coverage was ~7000X. Mutation Frequency (MF) is the proportion of reads containing the mutated allele compared with the total number of reads at that genomic position (Alt Cov/(Ref Cov+AltCov)). This data was adjusted for chromosome copy number to infer clonal size. Estimated sensitivity (Threshold) in detecting minor subclones was calculated independently for each mutation, considering the general occurrence of specific base substitutions/*Ins/Dels* in each sample. When MF is > threshold of sensitivity, the mutation is considered true, otherwise it is considered a sequencing artefact. The complete output of targeted deep sequencing with the estimated sensitivity is reported in Supplementary Table 3.

Supplementary Table S1.1: novel high quality somatic SNVs and *Ins/Dels* detected by WES in patient AML#1

Gene	Exon	cDNA	Tot Cov at diagnosis	MF at diagnosis	Tot Cov at relapse	MF at relapse	Provean Prediction	Sift Prediction
ATXN7L1	exon10	c.1977_1979del	38	0,1316	36	0,0000	Neutral	Deletion
NAP1L2	exon1	c.663_665del	26	0,1923	24	0,1250	Neutral	Deletion
KIF1A	exon29	c.G3010A	46	0,1957	34	0,0588	Neutral	Tolerated
TYK2	exon18	c.T2597A	95	0,3368	107	0,1495	Deleterious	Damaging
ISOC2	exon4	c.378delG	26	0,0000	18	0,2778	Frameshift	Frameshift
H2AFV	exon5	c.T356C	50	0,0400	28	0,2143	Deleterious	Tolerated

Tot Cov: total coverage, MF: mutation frequency, NA: not available.

Supplementary Table S1.2: novel high quality somatic SNVs and *Ins/Dels* detected by WES in patient AML#2

Gene	Exon	cDNA	Tot Cov at diagnosis	MF at diagnosis	Tot Cov at relapse	MF at relapse	Provean Prediction	Sift Prediction
EMX1	exon1	c.C454A	15	0,3333	39	0,0000	Neutral	Damaging
KMT2D	exon39	c.11580_11582del	25	0,2400	35	0,0000	Neutral	Deletion
GLRB	exon10	c.C1414T	36	0,5556	45	0,3556	Nonsense	Nonsense
SETD2	exon15	c.6306_6307insCACC	23	0,3478	42	0,4048	Frameshift	Frameshift
SRSF7	exon8	c.G677A	71	0,1972	71	0,2394	Neutral	Damaging
LRP1B	exon46	c.C7535T	44	0,2500	34	0,5588	Neutral	Tolerated
CEBPA	exon1	c.937_938insCAG	99	0,8990	103	0,7961	Deleterious	Insertion
DOCK8	exon2	c.C141A	112	0,4018	109	0,2018	Neutral	Tolerated
ZNF503	exon1	c.78_79insAGGCGGAGGCGG	51	0,2745	64	0,1563	Neutral	Insertion
FLRT1	exon2	c.G446A	70	0,4857	95	0,5474	Neutral	Damaging
RREB1	exon10	c.G3088A	13	0,8462	21	0,5714	Neutral	Damaging
BAIAP2	exon8	c.G805A	14	0,7143	36	0,4167	Neutral	Damaging
FAM46A	exon2	c.117_131del	13	0,3846	20	0,3000	Neutral	Deletion
UFL1	exon17	c.G1985A	24	0,0000	25	0,5600	Neutral	Tolerated
MAP1A	exon4	c.G163C	61	0,0000	95	0,1895	Deleterious	Damaging
DNAH14	exon75	c.11920_11923del	75	0,0000	87	0,3333	Frameshift	Frameshift
WISP1	exon5	c.G1040A	32	0,0000	28	0,3214	Neutral	Tolerated
FLVCR2	exon1	c.G103T	100	0,0000	160	0,3188	Neutral	Tolerated
LDLR	exon15	c.C2174G	144	0,0000	175	0,2571	Deleterious	Damaging
FAM73A	exon10	c.G1175T	75	0,0000	109	0,2294	Deleterious	Damaging
SSPO	exon2	c.G215A	20	0,0000	41	0,4634	NA	NA
SELP	exon3	c.G170A	88	0,0000	56	0,1429	Deleterious	Damaging
KCNAB3	exon1	c.G179A	37	0,0000	39	0,3590	Neutral	Tolerated
MIB1	exon13	c.G1862C	26	0,0000	26	0,3077	Deleterious	Damaging
TAS1R2	exon2	c.G406A	63	0,0000	49	0,1429	Neutral	Damaging
WT1	exon5	c.G962C	75	0,0000	15	0,4000	Neutral	Tolerated
ASXL3	exon12	c.C4409T	44	0,0000	49	0,2449	Deleterious	Damaging
ZNF671	exon4	c.C681A	44	0,0000	53	0,2642	Neutral	Tolerated
ZNF630	exon5	c.T346A	20	0,0000	51	0,1765	Neutral	Tolerated
TEX11	exon12	c.C881A	8	0,0000	26	0,3462	Deleterious	Damaging
IGSF21	exon4	c.T346A	45	0,0000	84	0,3333	Deleterious	Damaging
PSIP1	exon11	c.C1003A	35	0,0000	27	0,3704	Neutral	Tolerated
FAM155B	exon1	c.54_56del	39	0,0256	27	0,1852	Neutral	Deletion
BHLHE41	exon5	c.C893T	14	0,0000	19	0,2632	Neutral	Tolerated
PCBP2	exon15	c.G1077C	76	0,0000	65	0,1538	Neutral	Tolerated
CHST6	exon3	c.C1019T	158	0,0000	114	0,5702	Neutral	Tolerated
SCAPER	exon10	c.T309A	37	0,0000	41	0,2439	Neutral	Tolerated
XKR9	exon4	c.G343A	50	0,0000	54	0,2407	Neutral	Tolerated
COL6A2	exon26	c.2410_2411insT	24	0,0000	43	0,2558	Frameshift	Frameshift
FAM193A	exon17	c.G2671T	23	0,0000	23	0,2609	Neutral	Tolerated
KRBOX4	exon6	c.G315A	40	0,0000	29	0,5862	Nonsense	Nonsense

Ref: reference; Alt: alteration; Tot Cov: total coverage, MF: mutationfrequency, NA: not available.

Supplementary Table S1.3: novel high quality somatic SNVs and *Ins/Dels* detected by WES in patient AML#3

Gene	Exon	cDNA	Tot Cov at diagnosis	MF at diagnosis	Tot Cov at relapse	MF at relapse	Provean Prediction	Sift Prediction
RIMS1	exon1	c.G89A	27	0,2963	21	0,0000	Neutral	Tolerated
OR10G2	exon1	c.C508T	18	0,3889	14	0,1429	Deleterious	Damaging
SCAF4	exon20	c.2831_2833del	46	0,1304	27	0,1111	Neutral	Deletion
WT1	exon7	c.1139_1140insTCTTGTAC	80	0,1625	95	0,0316	Frameshift	Frameshift
ENAH	exon5	c.651_668del	79	0,1519	97	0,1237	Neutral	Deletion
FLT3	exon20	c.T2505A	48	0,0000	67	0,1791	Deleterious	Damaging
SUPT6H	exon34	c.4573_4575del	38	0,0000	40	0,1500	Neutral	Deletion
SALL1	exon2	c.T1517C	97	0,0000	103	0,2621	Deleterious	Damaging
PTPN11	exon3	c.C215T	51	0,0000	40	0,2750	Deleterious	Damaging
UBE2D3	exon6	c.A211G	16	0,0000	24	0,3333	Deleterious	Damaging
SOX11	exon1	c.673_675del	31	0,0323	45	0,1778	Neutral	Deletion

Ref: reference; Alt: alteration; Tot Cov: total coverage, MF: mutationfrequency, NA: not available.

Supplementary Table S1.4: novel high quality somatic SNVs and *Ins/Dels* detected by WES in patient AML#4

Gene	Exon	cDNA	Tot Cov at diagnosis	MF at diagnosis	Tot Cov at relapse	MF at relapse	Provean Prediction	Sift Prediction
CUZD1	exon2	c.A214T	25	0,3600	29	0,0000	Neutral	Damaging
WT1	exon7	c.1145_1146insTCGG	107	0,2150	141	0,0000	Frameshift	Frameshift
PYCR1	exon3	c.C285G	104	0,3654	144	0,0000	Deleterious	Damaging
TUBA3C	exon4	c.G1040T	15	0,3333	19	0,1053	Deleterious	NA
GPR132	exon5	c.A817G	50	0,0000	73	0,1918	Neutral	Tolerated
TEK	exon17	c.G2849A	35	0,0000	60	0,2167	Neutral	Tolerated
DLX2	exon3	c.G817T	16	0,0625	30	0,2000	Neutral	Tolerated

Ref: reference; Alt: alteration; Tot Cov: total coverage, MF: mutation frequency, NA: not available.

Supplementary Table S2: primers used for targeted deep sequencing on MiSeq System (Illumina)

Gene		Primer	Amplicon lenght	Annealing temperature (°C)	Patient
TYK2	F	AGAAATTGGCACACACCCTGA	343	60.13	AML#1
	R	TTGACTCTGCCTCTTGGGGA		60.47	
SETD2	F	GTGTGCAAGACAAGCTGAGTAA	436	60.34	AML#2
	R	AGCACCTTTCCAGCATTAGGG		59.13	
PSIP1	F	TTATGCTACGGAACTGCACCA	480	59.72	AML#2
	R	AGCATCCAGCTTTCAGCAAGT		60.55	
RREB1	F	GGGACAAGGATTTGGCCACT	541	60.25	AML#2
	R	GACTCTTTTGGGCTGGTGGT		60.18	
WISP1	F	AAGGTGGAATGCTCCCACATAG	521	60.09	AML#2
	R	AGGGTCCAGATCAGGGTAACT		59.63	
CEBPA	F	TCGGTGGACAAGAACAGCA	443	59.17	AML#2
	R	TCTTAGACGCACCAAGTCCG		59.76	
WT1	F	TGGGACTGGGGACTTAGTTCA	415	60.13	AML#2
	R	TTCCAGGGGCATGTTGATGAG		60.34	
ASXL3	F	GCCTCGAAACAGGGCAGATA	307	59,82	AML#2
	R	TGCCGAAGCAGCAATGAAAG		59,76	
FLT3	F	ACCTCCTACTGAAGTTGAGTCT	294	57.88	AML#3
	R	ACAGTGAGTGCAGTTGTTTACC		59.06	
PTPN11*	F	CGACGTGGAAGATGAGATCTGA	384	59.64	AML#3
	R	CAGTCACAAGCCTTTGGAGTCAG		61.60	
UBE2D3	F	TTGCTTCCCCATCTCTTACCAA	381	59.35	AML#3
	R	CAGGGGGTTGTGGTCAGATT		59.59	
SALL1	F	GACAGTGCCTTGCAGATCCA	202	60.32	AML#3
	R	ATGGGATGCCAGTACTCGTG		59.53	
WT1	F	TCTGGAGTGTGAATGGGAGTG	360	59.37	AML#3
	R	TGTGAGAGCCTGGAAAAGGAG		59.65	AML#4
TEK	F	AACCTTCTGGACTTCCTTCGC	203	60,27	AML#4
	R	AGAGGGAACTCCACAGGAAAGA		60,43	

F: forward primer; R:reverse primer.
*These primers were adopted from Tartaglia M. et al. “PTPN11 mutations in Noonan syndrome: molecular spectrum, genotype-phenotype correlation, and phenotypic heterogeneity.”*Am. J. Hum. Genet*. **70**, 1555–1563 (2002).

Supplementary Table S3: Complete targeted deep sequencing output

Gene	Chr	Start	End	Ref	Alt	Patient	Sample	Ref Cov	Alt Cov	MF	Threshold
TYK2	19	10467264	10467264	A	T	AML#1	Diagnosis	101107	76273	0,4300	0,0021
							Remission	86116	344	0,0040	0,0014
							Relapse	81803	14345	0,1492	0,0024
RREB1	6	7231420	7231420	G	A	AML#2	Diagnosis	19133	25956	0,5757	0,0044
							Remission	55541	112	ND	0,0037
							Relapse	51743	58650	0,5313	0,0044
PSIP1	9	15469966	15469966	G	T	AML#2	Diagnosis	23054	59	ND	0,0049
							Remission	118176	230	ND	0,0041
							Relapse	81299	63556	0,4388	0,0050
ASXL3	18	31324221	31324221	C	T	AML#2	Diagnosis	64939	188	0,0029	0,0028
							Remission	180435	371	ND	0,0022
							Relapse	140912	59561	0,2971	0,0026
WT1	11	32438075	32438075	C	G	AML#2	Diagnosis	264476	314	ND	0,0017
							Remission	526427	548	ND	0,0016
							Relapse	203969	136051	0,4001	0,0020
CEBPA	19	33792384	33792384	0	CTG	AML#2	Diagnosis	5715	44378	0,8859	0,0010
							Remission	127121	98	0,0008	0,0002
							Relapse	28268	119008	0,8081	0,0014
SETD2	3	47098968	47098968	0	GGTG	AML#2	Diagnosis	34660	16686	0,3250	0,0010
							Remission	208724	155	0,0007	0,0002
							Relapse	107784	50004	0,3169	0,0014
WISP1	8	134239889	134239889	G	A	AML#2	Diagnosis	88355	106	ND	0,0044
							Remission	172551	236	ND	0,0037
							Relapse	138467	71389	0,3402	0,0044
FLT3	13	28592640	28592640	A	T	AML#3	Diagnosis	208772	7397	0,0342	0,0021
							Remission	197604	62	ND	0,0014
							Relapse	158767	24390	0,1332	0,0024
WT1	11	32417913	32417913	0	GTACAAGA	AML#3	Diagnosis	241057	38837	0,1388	0,0006
							Remission	210264	0	ND	0,0007
							Relapse	144182	6313	0,0419	0,0007
SALL1	16	51174616	51174616	A	G	AML#3	Diagnosis	92946	288	ND	0,0057
							Remission	79237	194	ND	0,0054
							Relapse	87761	35069	0,2855	0,0078
UBE2D3	4	103722704	103722704	T	C	AML#3	Diagnosis	42854	140	ND	0,0056
							Remission	27734	59	ND	0,0050
							Relapse	2034	778	0,2767	0,0058
PTPN11	12	112888199	112888199	C	T	AML#3	Diagnosis	318663	408	ND	0,0031
							Remission	244880	310	ND	0,0028
							Relapse	160196	74902	0,3186	0,0036
TEK	9	27212867	27212867	G	A	AML#4	Diagnosis	48959	121	ND	0,0044
							Remission	91402	237	ND	0,0037
							Relapse	80300	21371	0,2102	0,0044
WT1	11	32417907	32417907	0	CCGA	AML#4	Diagnosis	220216	84098	0,2764	0,0010
							Remission	480887	98	ND	0,0002
							Relapse	360081	2	ND	0,0014

Chr: chromosome; Ref: reference; Alt: alteration; Cov: coverage; MF: mutation frequency; ND: not detected. MF is the ratio between Alt Cov and (Ref Cov+AltCov). When MF is > threshold of sensitivity, the mutation is considered true, otherwise it is considered a sequencing artefact.
